# Emissions of *Escherichia coli* Carrying Extended-Spectrum β-Lactamase Resistance from Pig Farms to the Surrounding Environment

**DOI:** 10.3390/ijerph120404203

**Published:** 2015-04-16

**Authors:** Lili Gao, Yeke Tan, Xiaodan Zhang, Jiaqing Hu, Zengmin Miao, Liangmeng Wei, Tongjie Chai

**Affiliations:** 1 College of Veterinary Medicine, Shandong Agricultural University, Tai’an 271000, Shandong, China; E-Mails: abigheart@163.com (L.G.); zhangxiaodan89@126.com (X.Z.); hujiaqing0609@126.com (J.H.); 2 Sino-German Cooperative Research Centre for Zoonosis of Animal Origin Shandong Province, Tai’an 271000, Shandong, China; 3 Key Laboratory of Animal Biotechnology and Disease Control and Prevention of Shandong Province, Tai’an 271000, Shandong, China; 4 Tai’an City Central Hospital, Tai’an 271000, Shandong, China; E-Mail: tykaaa@163.com; 5 College of Life Sciences, Taishan Medical University, Tai’an 271000, China; E-Mail: Zengminmiao@163.com

**Keywords:** ESBL-producing *E. coli*, environment, emission, CTX-M

## Abstract

The dissemination of extended-spectrum β-lactamase (ESBL)-producing *Escherichia coli* (*E. coli*) from food-producing animals to the surrounding environment has attracted much attention. To determine the emissions of ESBL-producing *E. coli* from pig farms to the surrounding environment, fecal and environmental samples from six pig farms were collected. In total, 119 ESBL-producing *E. coli* were isolated from feces, air samples, water, sludge and soil samples. Antibiotic susceptibility testing showed that the ESBL-producing isolates were resistant to multiple antibiotics and isolates of different origin within the same farm showed similar resistance phenotypes. Both CTX-M and TEM ESBL-encoding genes were detected in these isolates. CTX-M-14 and CTX-M-15 were the predominant ESBL genes identified. ESBL producers from feces and environmental samples within the same farm carried similar CTX-M types. The results indicated that the ESBL-producing *E. coli* carrying multidrug resistance could readily disseminate to the surrounding environment.

## 1. Introduction

The increasing prevalence of extended-spectrum β-lactamases (ESBLs) in the World has attracted wide attention [1]. ESBLs are enzymes that can destroy β-lactam antibiotics, including penicillins, first, second and third-generation cephalosporins and aztreonam which are susceptible to β-lactamase inhibitors [2]. The ability of ESBLs to confer bacterial resistance can dramatically decrease therapeutic options in disease control and treatment [3,4]. The ESBL enzymes mainly include three types, TEM, SHV and CTX-M [5]. Since first discovered in 1989 [6], CTX-M gene has become the most common ESBL enzyme and has spread quickly throughout the World taking the place of TEM and SHV types that were prevalent in the early 1990s [5]. Genes encoding these various ESBL genes are located on mobile genetic elements and could disseminate through horizontal gene transfer between bacteria, and even between different species [7]. 

Food-producing animals were considered reservoirs of zoonotic pathogens and resistant bacteria [8]. *Escherichia coli* can survive in the gastrointestinal tract of food-producing animals as a commensal bacterium and can also cause infections [9]. Extended-spectrum cephalosporins are effective drugs against such infections in veterinary clinical use; which creates a selective pressure for ESBL-producing *E. coli*. Animals colonized with ESBL-producing *E. coli* have been considered as potential sources of resistant *E. coli* infections in the community, which has attracted wide concern [10]. Furthermore; the ESBL-producing *E. coli* in animal farms could influence public health through environment pollution and contaminated animal products [11].

The dissemination of these resistant bacteria from animal houses through various routes exerts pressure on the surrounding environment and even influences the living environment of human beings. Aerosol transmission is an important route for virus and bacteria [12]. *E. coli* has been identified to transmit through air by aerosol formation [13]. Aerosol transmission of ESBL-producing *E. coli* with air flow contributes to its dissemination. The discharge of waste products and farmland application of effluents and feces could also promote the entry of drug-resistant bacteria into the environment [14]. To date, ESBL-producing bacteria have been found in various environments, where they may be a reservoir contributing to the spread of resistant bacteria [14,15]. In this study, to estimate the transmission of ESBL-producing *E. coli* originated from pig farms to the surrounding environment, ESBL-producing *E. coli* was collected from fecal and environmental samples from pig farms in China.

## 2. Materials and Methods

### 2.1. Pig Farms

Six pig farms located in different regions of Shandong Province, China and their surrounding environments were selected to collect samples to investigate the transmission of ESBL-producing *E. coli* from food animal-producing houses to the surrounding environment. ESBL-producing *E. coli* has been found in six (A, B, C, D, E and F) out of ten pig farms in our primary research. These farms are far away from villages. Negative pressure ventilation was used in these farms.

### 2.2. Sampling

Fecal and environmental samples were collected from these farms between April 2013 and June 2013 to evaluate the spread of resistant bacteria produced in pig farms to the surrounding environment. Air samples were collected using a six-stage Anderson sampler [16] at an airflow rate of 28.3 L/min placed at a height of 1.0 m indoors and outdoors in the down- and upwind positions as previously reported [13]. Each time, six MacConkey agar plates with 2 µg/mL cefotaxime were used as medium placed in an Anderson six-stage sampler for air sample collection. Inside and outside air samples were collected at the same time. In each house, inside air samples were collected at three locations along the passage with a time of 20–30 min. Outside air samples were collected at different distances including 10 m and 50 m upwind, and 10 m, 50 m, and 100 m downwind. No air samples were collected from farms E and F. 

At the same time, environmental samples were collected. Water and sludge were collected in the vicinity of pig farms A, B, C and D. River water samples were collected at 10 m upstream, and 10 m, 50 m and 100 m downstream away from the drain outlet. Sludge samples were collected at the outlet of the effluent. Soil samples were collected at different directions outside of the animal house. These samples were transferred to an ice box and then processed immediately upon arrival at the lab.

### 2.3. Cefotaxime-Resistant E. coli Isolation

After collection, the MacConkey agar plates with 2 µg/mL cefotaxime used for air samples were incubated at 37 °C overnight directly. Fecal samples were serially diluted twice with sterile phosphate buffered saline solution and then 100 µL was cultured on MacConkey agar plates with 2 µg/mL cefotaxime and incubated overnight. The river water samples were filtered using a nitrocellulose membrane filter and then the filter was placed on agar plates. Soil and sludge samples (2 g) were transferred into 50 mL Luria-Bertani (LB) broth for enrichment. Following bacteria enrichment, overnight cultures were streaked on MacConkey agar plates with 2 µg/mL cefotaxime at 37 °C overnight. One or two colonies with typical *E. coli* morphology were selected and further streaked on LB agar plates for purification. Presumptive pure cultures were identified by classical biochemical methods and the API 20E system [17]. 

### 2.4. Confirmation and Antimicrobial Susceptibility Testing

The *E. coli* isolates from feces and environmental samples were subjected to a double disk diffusion method for confirmation of the ESBL-producing *E. coli* using ceftazidime or cefotaxime, alone or together with clavulanic acid. The antimicrobial susceptibility of the *E. coli* isolates was tested by the disk diffusion method on Mueller-Hinton agar plates using the following antibiotics: ampicillin (AMP, 10 µg), amoxicillin/clavulanic acid (AMC, 20 µg + 10 µg), piperacillin/tazobactam (TZP, 100 µg + 10 µg), ampicillin/salbactam (SAM, 10 µg + 10 µg), cephalothin (CF, 30 µg), cefuroxime (CXM, 30 µg), aztreonam (ATM, 30 µg), ciprofloxacin (CIP, 5 µg), norfloxacin (NOR, 10 µg), gentamicin (GM, 10 µg), tetracycline (TE, 30 µg), streptomycin (S, 10 µg), chloramphenicol (C, 30 µg), kanamycin (K, 30 µg), nalidixic acid (NA, 30 µg), trimethoprim/sulfamethoxazole (SXT, 25 µg) and trimethoprim (TMP, 5 µg) according to CLSI [18]. The *E. coli* ATCC 25922 was used for quality control.

### 2.5. Resistance Genes

TEM-, SHV-, and CTX-M-encoding ESBL genes were identified using multiplex polymerase chain reactions (PCR) to determine the ESBL types of the ESBL producing *E. coli* from different samples, as previously described [19]. TEM-encoding genes were further amplified as described previously [20] and the amplicons were sequenced. The *bla*_CTX-M_ genes were further amplified and analyzed using group primers CTX-M-1, CTX-M-2, CTX-M-8 and CTX-M-9, as described previously [21,22]. The PCR products were purified and cloned in pMD-18T for sequencing. The obtained DNA sequences were compared and blasted (http://www.ncbi.nlm.nih.gov/) to confirm the β**-**lactamase gene subtype. 

### 2.6. Statistical Analysis 

Pearson’s chi-square test was used to compare the continuous data. The association between resistance phenotype of isolates from fecal and environmental samples were evaluated. Correlation coefficients (r values) and the levels of significance (*p* values) were used to interpret the results of correlation analyses. Two-tailed p values of 0.05 were considered statistically significant. The statistical analyses were conducted using the statistics software, SPSS, version 19.0 (IBM SPSS, Chicago, IL, USA).

## 3. Results

### 3.1. Samples Positive for ESBL-Producing E. coli from Feces and Environments

One hundred and twenty samples positive for ESBL-producing *E. coli* were detected from the fecal samples from the six farms. Water and sludge samples were collected from A, B, C and D farms. In the vicinity of E and F farm, no river was found. Samples positive for ESBL producers were detected in air samples in three out of four pig farms. 

ESBL positive water samples were found in all the four pig farms with collected water samples. Of the sludge samples, samples positive for ESBL-producing E. coli were found in farm A, B and D. Soil samples were collected from all six farms. On farm E, planting soil samples were also collected as the soil was amended with feces from the farm. ESBL-positive samples were only found in planting soil samples from farm E and two from surface soil in farm F (Table 1).

**Table 1:** No. of samples (total) and ESBL-positive samples (+) from six pig farms.

Farms	No of Feces		No. of Air Samples		No. of Water Samples		No. of Sludge Samples		Soil Samples	
	Total	+	Total	+	Total	+	Total	+	Total	+
A	30	5	8	0	10	1	5	2	10	0
B	30	10	8	3/3	10	2	5	2	10	0
C	30	10	8	1	10	3	5	0	10	0
D	30	16	8	2	10	2	5	1	10	0
E	40	32	-	-	-	-	-	-	80	10
F	40	13	-	-	-	-	-	-	20	2

### 3.2. Isolation and Identification of ESBL-Producing E. coli

A total of 120 cefotaxime-resistant *E. coli* strains were isolated from fecal samples and environmental samples collected from the six pig farms. One hundred and nineteen *E. coli* isolates from feces, indoor air samples and outdoor air samples, water and sludge samples and soil samples were confirmed to be ESBL-producing *E. coli* after the phenotypic confirmatory test. In three out of six pig farms (B, C and D), ESBL-producing *E. coli* were detected in air samples (six, one and two, respectively). Seven ESBL-producing *E. coli* were obtained from water samples outside the four farms, and five from sludge samples. A total of 12 ESBL-producers were isolated from soil samples.

From farm A, five isolates were obtained from feces, one came from water (10 m downstream), and two were isolated from sludge samples. Among the 20 ESBL-producing *E. coli* isolated from farm B, 10 isolates were from feces, six were from air samples including three indoor air isolates and three outdoor air isolates from 10 m and 100 m downwind, and four were from water (10 m downstream) and sludge samples. In farm C, two water isolates from 10 m downstream and one from 50 m downstream, and one ESBL-producing isolates from an indoor air sample, outdoor air sample (10 m downwind), water and sludge sample respectively were obtained (Table 2).

**Table 2:** No. of 119 ESBL-producing *E. coli* and their locations.

Farm	Samples	No. of ESBL Producers	Locations
Farm A	Feces	5	
	Water samples	1	10 m downstream
	Sludge samples	2	
Farm B	Feces	10	
	Indoor air samples	3	
	Outdoor air samples	3	10 m downwind
			100 m downwind
	Water samples	2	10 m downstream
	Sludge samples	2	
Farm C	Feces	10	
	Indoor air samples	1	
	Water samples	3	10 m downstream
			50 m downstream
Farm D	Feces	16	
	Indoor air samples	1	
	Outdoor air samples	1	10 m downwind
	Water samples	1	10 m downstream
	Sludge samples	1	
Farm E	Feces	32	
	Soil samples	10	Amended soil
Farm F	Feces	13	
	Soil samples	2	

### 3.3. Antimicrobial Susceptibility Testing

All the ESBL-producing *E. coli* from the six pig farms were susceptible to AMC, TZP, SAM and TMP, but resistant to AMP and CF, and highly resistant to PRL and TE. Isolates from B, D and F were all resistant to ATM. For other antibiotics, different resistance phenotypes were obtained from these ESBL producers between these six farms. High resistance to GM, S and K was observed in farms B, D, E and F. The ESBL-producers from different farms also showed different resistance levels to CIP, NA, SXT and C. Significant associations in resistance rate were observed between isolates from feces and environmental samples within the same farm (Table 3).

**Table 3:** Resistance to 17 antibiotics of the 119 ESBL-producing *E. coli* isolates. Fecal isolates, FI; Environmental isolates, EI. *p* < 0.01.

Antibiotics	Farm A		Farm B		Farm C		Farm D		Farm E		Farm F	
	(n = 8)		(n = 20)		(n = 14)		(n = 20)		(n = 42)		(n = 15)	
	FI	EI	FI	EI	FI	EI	FI	EI	FI	EI	FI	EI
AMP	100	100	100	100	100	100	100	100	100	100	100	100
CF	100	100	100	100	100	100	100	100	100	100	100	100
PRL	100	67	50	30	90	75	63	50	94	80	85	100
CXM	60	100	80	80	30	0	100	75	100	100	92	100
ATM	0	0	100	100	0	0	100	100	94	80	100	100
AMC	0	0	0	0	0	0	0	0	0	0	0	0
TZP	0	0	0	0	0	0	0	0	0	0	0	0
SAM	0	0	0	0	0	0	0	0	0	0	0	0
GM	0	0	70	60	30	25	63	75	44	60	85	50
K	20	0	90	70	10	0	100	75	69	70	92	100
S	0	0	60	60	40	75	63	50	81	70	54	50
TMP	0	0	0	0	0	0	0	0	0	0	0	0
TE	60	33	100	80	90	75	100	100	91	90	85	0
CIP	20	0	70	80	10	0	44	50	53	10	23	0
NA	80	67	90	80	40	25	81	75	56	10	23	0
SXT	0	33	100	80	80	100	56	50	84	60	92	100
C	20	33	70	60	90	100	88	75	91	70	100	0
r	0.892		0.971		0.944		0.969		0.914		0.752	

### 3.4. ESBL Gene

TEM and CTX-M genes were detected in all ESBL-producing isolates from the six pig farms. However, no SHV gene was found. In these pig farms, five kinds of CTX-M subtypes were detected including *bla*_CTX-M-14_, *bla*_CTX-M-15_, *bla*_CTX-M-24_, *bla*_CTX-M-27_ and *bla*_CTX-M-65_. The diversity of CTX-M genes of ESBL-producing isolates from feces and environmental samples tended to be similar, however, the prevalent CTX-M types and kinds of subtypes varied between farms. 

In farm A, only *bla*_CTX-M-15_ was detected. *Bla*_CTX-M-14_ was the predominant CTX-M-encoding gene in farm B (n = 7), followed by *bla*_CTX-M-24_ (n = 3), *bla*_CTX-M-15_ (n = 2), *bla*_CTX-M-27_ (n = 2), and *bla*_CTX-M-65_ (n = 1). *Bla*_CTX-M-14_, *bla*_CTX-M-15_, *bla*_CTX-M-24_, and *bla*_CTX-M-27_ were both detected in fecal and environment original ESBL-producing *E. coli*. In farm C, *bla*_CTX-M-14_ and *bla*_CTX-M-15_ were both detected in fecal and environmental isolates except *bla*_CTX-M-27_. Among the three kinds of *bla*_CTX-M_ detected, *bla*_CTX-M-14_ was the predominant CTX-M type that both detected in feces and environmental samples. In farms E and F, the predominant CTX-M type was *bla*_CTX-M-15_, followed by *bla*_CTX-M-14_ in fecal and environmental samples. *Bla*_CTX-M-27_ and *bla*_CTX-M-65_ were also detected farm E, but *bla*_CTX-M-65_ was not found in farm F (Table 4).

**Table 4:** β-Lactamase genes in the phenotypic detected ESBL-producing *E. coli* from fecal and environmental samples. Fecal isolates, FI; Environmental isolates, EI.

Genes	Farm A		Farm B		Farm C		Farm D		Farm E		Farm F	
	(n = 8)		(n = 20)		(n = 14)		(n = 20)		(n = 42)		(n = 15)	
	FI	EI	FI	EI	FI	EI	FI	EI	FI	EI	FI	EI
CTX-M-14	-	-	6	1	4	2	6	1	5	3	3	1
CTX-M-15	4	2	1	1	-	-	-	-	12	3	7	1
CTX-M-24	-	-	1	2	-	-	-	-	-	-	-	-
CTX-M-27	-	-	1	1	3	-	2	-	7	-	2	-
CTX-M-65	-	-	-	1	2	1	2	1	7	1	-	-
CTX-M	4	2	9	6	9	3	10	2	31	7	12	2
CTX-M/TEM	3	2	4	4	5	0	4	0	26	6	8	1
TEM-1	4	3	5	8	6	1	10	2	26	9	9	1

## 4. Discussion

With the use of antibiotics, more and more resistant bacteria occur in food-producing animals, including ESBL-producing *E. coli*. The spread of these bacteria through various routes to the environment creates a threat to public health. In this study, ESBL-producing *E. coli* was isolated from feces and environment samples including indoor air, outdoor air, water and sludge samples and soil samples from six pig farms in rural regions of Shandong, China. From the six farms, ESBL-producing *E. coli* was all detected in feces and different kinds of environmental samples, which indicated the possible transmission routes of ESBL-producers from food-producing animal farms.

All 119 of the ESBL-producing isolates from fecal and environmental samples showed high rates of resistance to multiple antimicrobial agents. Isolates showing resistance to two or more classes of drugs were treated as multi-drug resistant (MDR). The resistance profiles varied between different farms, but were highly related between isolates from feces and environmental samples within the same farm. These results suggested that the ESBL-producers in the environment might originate from the pig farm.

The CTX-M gene was the predominant ESBL gene in this region, consistent with previous reports [23]. *Bla*_CTX-M-14_ and *bla*_CTX-M-15_ were the most common CTX-M type, similar to what has been reported in pigs, cattle, and chickens [11,24]. Various CTX-M subtypes were detected, including *bla*_CTX-M-14_, *bla*_CTX-M-15_, *bla*_CTX-M-24_, *bla*_CTX-M-27_ and *bla*_CTX-M-65_. The ESBL-producing isolates from feces and environmental samples within the same farm tend to carry the same kind of CTX-M gene, while the diversity of CTX-M subtypes varied between different pig farms. 

In food-producing animal production, high concentrations of airborne microorganisms are often found in indoor environments [25]. These microbes in such an environment can survive in the form of aerosols for a long time in the air and transmit with air flow [13]. In this study, ESBL-producing *E. coli* was obtained from the indoor air and outdoor air samples. Isolates from indoor, outdoor and fecal samples showed high similarity, which indicated the airborne transmission of the ESBL-producing *E. coli* in pig farms. Previous studies had demonstrated the dissemination of ESBL-producing *E. coli* originated from chicken houses into the air [26,27]. The concentrations of microorganisms were closely related with the air quality. A poor air environment could benefit the spread of ESBL-producing *E. coli*. 

ESBL-producing bacteria have been increasingly reported in water and sludge [28,29,30]. Agricultural use of contaminated water or sludge could be a possible route for ESBL-producing *E. coli* to enter into the food chain [31,32]. In this study, ESBL-producing *E. coli* were also isolated from river water and sludge samples, which shared similar resistance profiles and ESBL genes with fecal isolates within the same farm. These results suggested the potential influence of pig farms on the surrounding water environments.

In conclusion, the high similarities of isolates from environmental and fecal samples suggest a possible dissemination of resistant bacteria from pig feces into the surrounding environment. These results indicated the emissions of resistant *E. coli* isolates from pig houses to the surrounding environment, which constitutes a major threat to public health. As the origin of resistant bacteria, thus the rational use and antibiotics and the establishment of effective management of food-producing animal farms are necessary.

## 5. Conclusions

Comparison of isolation rates, resistance profiles and β-lactamase genes showed that fecal isolates and environmental isolates shared similar characteristics, which suggested the possible emissions of the ESBL-producing *E. coli* from feces to the environment. 
